# Dynamics of Soluble Factors and Double-Negative T Cells Associated with Response to Renal Denervation in Resistant Hypertension Patients

**DOI:** 10.3390/jpm12030343

**Published:** 2022-02-24

**Authors:** Joana Delgado-Silva, Paulo Rodrigues-Santos, Jani-Sofia Almeida, Manuel Santos-Rosa, Lino Gonçalves

**Affiliations:** 1 Faculty of Medicine (FMUC), University of Coimbra, 3000-370 Coimbra, Portugal; lgoncalv@ci.uc.pt; 2 Department of Cardiology, Coimbra’s Hospital and University Centre, 3004-561 Coimbra, Portugal; 3 Coimbra Academic Clinic Centre (CACC), 3004-531 Coimbra, Portugal; paulo.santos@fmed.uc.pt (P.R.-S.); janisofiaalmeida@hotmail.com (J.-S.A.); msrosa@fmed.uc.pt (M.S.-R.); 4 Cardiovascular Intervention Unit (UNIC), Coimbra’s Hospital and University Center, 3004-561 Coimbra, Portugal; 5 Laboratory of Immunology and Oncology, Center for Neuroscience and Cell Biology (CNC), University of Coimbra, 3004-504 Coimbra, Portugal; 6 Center of Investigation in Environment, Genetics and Oncobiology (CIMAGO), Faculty of Medicine, University of Coimbra, 3000-370 Coimbra, Portugal; 7 Coimbra Institute for Clinical and Biomedical Research (iCBR), Faculty of Medicine, University of Coimbra, 3000-548 Coimbra, Portugal; 8 Center for Innovation in Biomedicine and Biotechnology (CIBB), University of Coimbra, 3000-548 Coimbra, Portugal

**Keywords:** hypertension, renal denervation, RANTES, cytokines, chemokines, immune response, inflammation

## Abstract

The role of the immune system, and hence inflammation, in the pathophysiology of hypertensive patients is not clear. Until now, most clinical and biochemical parameters have failed to predict a positive response to renal denervation (RDN). Our aim was to evaluate the immune response in a cohort of patients treated by RDN, through the analysis of cytokine, chemokine, and growth factor behavior. A population of 21 resistant hypertension patients, treated by RDN, was evaluated at six months and one year. Response was defined as a drop of ≥5 mmHg in ambulatory blood pressure monitoring. Sixty-seven percent and 81% of patients clinically responded after six months and one year, respectively. There were no complications or safety issues. Plasmatic levels of 45 cytokine, chemokine, and growth factors were quantified at four different times, pre- and post-procedure. Baseline characteristics were similar between groups, except that active smoking was more frequent in non-responders at one year. Regulated on activation, normal T cell expressed, and secreted (RANTES/CCL5) levels were significantly lower in responders, both at baseline and at 30 days (*p* = 0.037), and a level ≤15,496 pg/mL was the optimal cutoff, for prediction of a response. IL-15, IL-17A, IL-27, and leukemia inhibitory factor varied significantly in time, with an acute rise being observed 24 h after RDN. Our group has previously showed that HLA-DR+ double-negative (DN) T cells were significantly lower in responders. There was a positive correlation between IL-13, -27, and -4, and DN T cells, and a negative correlation between the latter and SDF-1α and TNF-α, at baseline. Low plasmatic levels of the chemokine RANTES/CCL5 was the most significant result associated with RDN response and may help to identify the best candidates among patients with true resistant hypertension. Pro-inflammatory cytokines correlated negatively with DN T cells in responders, a finding compatible with an enhanced inflammatory milieu present in this extremely high cardiovascular risk cohort.

## 1. Introduction

The identification of specific patient subsets who derive the most benefit from renal denervation (RDN) has been the focus of investigators in recent years. Following the unexpected outcomes of HTN-3 trial [1], three second generation randomized trials on RDN [2,3,4] were carefully constructed and have shown a significant decrease of blood pressure (BP) in a wider cohort of patients, with and without anti-hypertensive drugs, demonstrating not only efficacy but also safety. The role of the immune system, and hence inflammation, in the pathophysiology of hypertensive patients treated with RDN is not clear and, until now, most clinical and biochemical parameters have failed to predict the response to RDN [5].

Several mechanisms contribute to the pathogenesis and perpetuation of hypertension (HT), in which the renin–angiotensin–aldosterone system (RAAS) has major implications. Angiotensin II binds to angiotensin 1 receptors (AT1), usually present in several immune cells such as T cells, dendritic cells, and macrophages, and determines their differentiation and subsequent pro-inflammatory cytokine production [6]. Pro-inflammatory stimuli trigger endothelial expression of adhesion molecules and increase leucocyte migration, promoting fibrosis and hypertrophy with reduction of vascular luminal diameters [7]. Additionally, angiotensin II contributes to inflammation as it increases reactive oxygen species (ROS) production (including superoxide), by stimulating nicotinamide adenine dinucleotide (NADH) and nicotinamide adenine dinucleotide phosphate (NADPH) oxidase, leading to reduced levels of nitric oxide (NO) and development of oxidative stress. Mitochondrial oxidative stress (the main source of ROS) may lead to both end-organ dysfunction and HT, by enhancing more NADPH production, the RAAS system, and pro-inflammatory cytokine secretion which, in turn, stimulate oxidase activity and superoxide production, creating a deleterious feedback process [8,9,10].

Dörr et al. aimed to study the impact of endothelial adhesion molecules, which are indicators of endothelial dysfunction, on RDN response and concluded that responders may be more affected by HT-related endothelial dysfunction and shear stress [11]. The same research group studied the impact of brain-derived neurotrophic factor (BDNF) on RDN response. BDNF is a neurotrophin directly involved in the regulation of neurotransmitter production by the sympathetic nervous system (SNS). The authors found a significant decrease in BDNF levels immediately after RDN that correlated with systolic BP reduction at 6-month follow-up (FU), which is in accordance with previous knowledge that a decrease in BDNF expression is associated with an impaired density of sympathetic activity and transmitter release [12]. Eikelis et al. assessed the bioavailability of NO in patients treated with RDN, hypothesizing that it could function as a potential mechanism to the BP lowering effects. In this study, none of the plasma cytokines measured had a predictive value to differentiate responders from non-responders but the authors found a significant reduction in soluble fms-like tyrosine kinase-1 (sFLT-1) and, interestingly, NO elevation was only seen in responders. A possible explanation for this is the disassembly of the vascular endothelial growth factor (VEGF)-NO pathway, which prevented generation of NO and therefore limited the BP lowering effect in non-responders [13].

Previously, our group has explored the cellular immune profile of patients with resistant HT and treated with RDN. Levels of HLA-DR+ double-negative (DN) T cells were significantly higher, both at baseline and post-procedure, in the non-responders, a finding that is aligned with current knowledge stating the presence of a more profuse inflammatory milieu in this group, which could contribute to the perpetuation and severity of HT [14]. As a continuation of this research, the aim of the current study was to evaluate the behavior of a subset of cytokines, chemokines and growth factors in a cohort of patients with resistant HT treated with RDN, and to establish whether there is any correlation between soluble factors and DN T cells before RDN.

## 2. Material and Methods

### 2.1. Patients

The study population in this prospective, non-randomized, single center study included 21 patients with resistant HT and treated with RDN between May 2014 and October 2017. Methods have been previously described in a preliminary analysis conducted by our team [14]. In summary, patients were included if they presented with idiopathic resistant HT (treated with three or more anti-hypertensive drugs at maximum tolerated dosages, including a diuretic), measured by ambulatory BP monitoring (ABPM) and presenting with a mean systolic BP > 135 mmHg. Patients with renal dysfunction (glomerular filtration rate < 45 mL/min/1.73 m^2^), recent major cardiovascular events, fibromuscular dysplasia, previous renal angioplasty, or untreated secondary HT were excluded. Basal assessment consisted of a routine hematology and biochemistry profile, ABPM monitoring, electrocardiogram, transthoracic echocardiogram, 24-h rhythm monitoring and renal doppler or CT scan. Adherence was assessed in all patients by witnessed drug-intake. Patients were considered ‘responders’ (R) if a drop of ≥5 mmHg in mean ambulatory BP was observed at six months (vs. NR). Additionally, ‘late responders’ included ‘responders’ at six months and patients who responded after one year FU (R1Y vs. NR1Y).

The study was approved by the Faculty of Medicine of the University of Coimbra and the Coimbra’s Hospital and University Centre Ethics Committees, and all patients signed an informed consent form.

### 2.2. Renal Denervation

Patients received peri-procedural analgesia and conscious sedation with propofol/midazolam and remifentanil intravenous perfusion. Anti-thrombotic treatment included aspirin and a bolus of weight-adjusted unfractioned heparin (70–100 U/Kg). Femoral arterial access was obtained with 6F or 8F sheaths. Radiofrequency energy was delivered using the single-tip radiofrequency Symplicity Flex catheter (Medtronic Inc., Santa Rosa, CA, USA), the EnligHTN system (St. Jude Medical, MN, USA), or the Symplicity Spyral catheter (Medtronic Inc., Santa Rosa, CA, USA) in 9.5%, 38.1%, and 52.4% of the patients, respectively. Energy was applied to the main artery and branches, when feasible and according to the characteristics of each device with the goal of achieving a maximum number of circumferential ablation points. Radiofrequency energy was delivered according to a programmed algorithm specific to each device. Administration of intra-renal nitrates and a final renal angiogram was performed. All procedures were conducted by one interventional cardiologist dedicated to the field. Hemostasis was accomplished with a vascular closure device. All patients were monitored for 24 h before discharge.

### 2.3. Follow-Up

Patients were evaluated at 7, 30, 90, 180, and 365 days after the procedure. A renal angiogram via the radial artery was performed at 180 days to assess safety. An electrocardiogram, transthoracic echocardiogram and ABPM were performed after six months and one year. Venous blood samples were obtained on the following four occasions: D0 (before RDN), D1 (24 h after RDN), D7 (one week after RDN), and D30 (one month after RDN).

### 2.4. Soluble Cytokine, Chemokine and Growth Factors Profiling

Plasma of RDN patients was freshly isolated and stored at −20 °C until thawing before analysis. Plasmatic levels of cytokines, chemokines, and growth factors were quantitatively analyzed by commercially available single well Luminex^®^ xMAP assay (ProcartaPlex, Invitrogen, Waltham, MA, USA). For this study we used a broad human factor panel consisting of 45 protein targets comprising the following five modular subpanels: Th1/Th2 [GM-CSF, IFN gamma, IL-1 beta, IL-2, IL-4, IL-5, IL-6, IL-12p70, IL-13, IL-18, TNF alpha], Th9/Th17/Th22/Treg [IL-9, IL-10, IL-17A (CTLA-8), IL-21, IL-22, IL-23, IL-27], Inflammatory cytokines [IFN alpha, IL-1 alpha, IL-1RA, IL-7, IL-15, IL-31, TNF beta], Chemokines [Eotaxin (CCL11), GRO alpha (CXCL1), IP-10 (CXCL10), MCP-1 (CCL2), MIP-1 alpha (CCL3), MIP-1 beta (CCL4), RANTES (CCL5), SDF-1 alpha] and Growth factors [BDNF, EGF, FGF-2, HGF, NGF beta, PDGF-BB, PlGF-1, SCF, VEGF-A, VEGF-D]. Ninety-six well plates were read in a Luminex^®^ 200 System (Luminex Corporation, Austin, TX, USA) and plasmatic concentrations were calculated by applying a five-parameter logistic (5PL) regression non-linear model using the Luminex xPONENT v3.1 software.

### 2.5. Statistical Analysis

Categorical variables were characterized by determining the absolute and relative frequencies and, for numerical variables, the means and standard deviations. Comparisons between groups of categorical variables were conducted using the Chi-square test. For continuous variables, the Mann Whitney U test was used to compare two groups and the Kruskal–Wallis test for comparisons between more than two groups. A general linear model for repeated measures was applied to analyze variance of each laboratorial parameter, measured several times on each subject from the two different groups, which were ‘responder’ and ‘non-responder’. Two different responder groups were considered, responders at six months and at one year.

Statistical analyses were conducted using SPSS 19.0^®^, at a 5% significance level for hypothesis testing. The Type I error probability associated with this test of this null hypothesis is 0.05.

## 3. Results

### 3.1. Baseline Characteristics and Response to Renal Denervation

Twenty-one patients were included in this study (mean age 59 ± 11.3 years, 33.3% female). Fourteen patients were ‘responders’ (R) at six months (66.7%), and 17 were ‘late-responders’ (R1Y; 81%). Data regarding the groups of non-responders (NR and NR1Y) have previously been published [14]. There were no significant differences between responders and non-responders regarding comorbidities such as dyslipidemia, type 2 diabetes or the presence of sleep apnea, but active smoking was more frequent in the group of non-responders at one year (NR1Y) (*p* = 0.008). The overall group was overweight (BMI 29.7 ± 3.9). Baseline mean office systolic and diastolic BP was 189.5 ± 26 and 106.1 ± 18.6 mmHg, and ABPM mean systolic and diastolic BP was 153.8 ± 12.2 and 89 ± 14.6 mmHg, respectively (*p* = ns). Of the patients in the R and R1Y groups, 28.6% and 41.2% were non-dipper (vs. 71.4% and 50% in NR and NR1Y, *p* = ns). An ABPM systolic BP mean drop of 21.1 ± 13.3 and 16.2 ± 16.6 mmHg was observed in the R and R1Y groups, respectively (vs. −7.6 ± 10.6 and −8.5 ± 11.9 mmHg in the non-responders) (*p* < 0.02). Baseline characteristics, number of ablations and BP changes during follow-up are shown in Table 1.

### 3.2. Low Levels of RANTES Are Associated with Response to Renal Denervation

**Figure 1:**
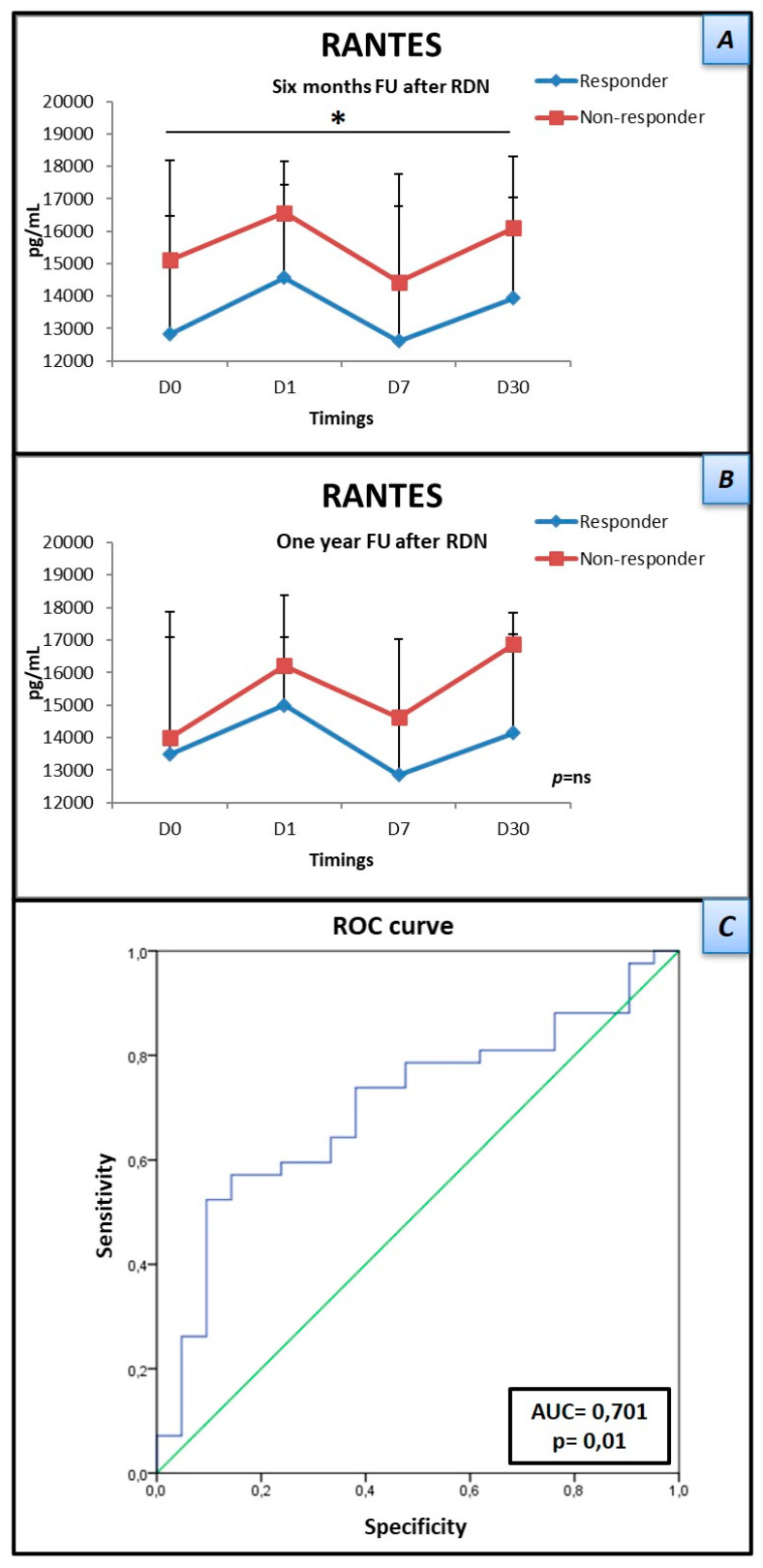
Quantification of RANTES at four time points (D0, D1, D7 and D30—see text), before and after renal denervation. (**A**) Plasmatic levels of RANTES are significantly lower in ‘responders’ (blue line) at six months. (**B**) After one year follow-up, levels of RANTES are still lower, even though statistically non-significant. (**C**) ROC curve: A RANTES cut-off value of ≤15,496 pg/mL showed the best overall sensitivity and specificity for determining renal denervation response. Data are presented as mean ± standard deviation. RANTES—regulated upon Activation, Normal T cell Expressed, and Secreted; FU—follow-up; RDN—renal denervation; AUC—area under the curve. * *p* < 0.05.

**Figure 2:**
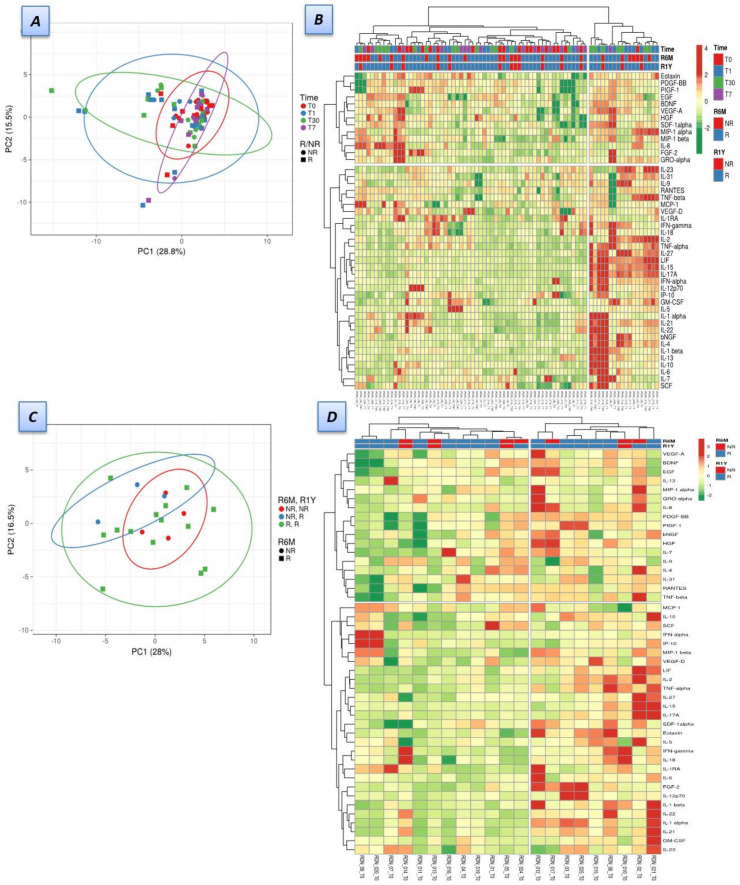
Distribution of cytokines, chemokines, and growth factors, according to renal denervation response, at six months and one year. (**A**) Principal component analysis for the distribution of cytokines, chemokines, and growth factors in R/NR and R1Y/NR1Y at the four analyzed time points (D0, D1, D7 and D30—see text). (**B**) Heatmap for plasmatic cytokines, chemokines, and growth factors in R/NR and R1Y/NR1Y, at the four time points. (**C**) Principal component analysis for the distribution of cytokines, chemokines, and growth factors in R/NR and R1Y/NR1Y, at baseline (**D**) Heatmap at baseline for cytokine, chemokine, and growth factor quantification in R/NR and R1Y/NR1Y. Bars at the top of the heatmaps represent each patient individualized (**B**,**D**), the time point of the analysis (**D**) and the groups according to response (R/NR—six months; R1Y/NR1Y—one year), in accordance with the legend on the right side of the figure. Numbers at the bottom of the heatmaps represent patient case number identifiers. RDN—renal denervation.

As described, absolute levels of 45 protein cytokines, chemokines, and growth factors were quantified at four different times, pre- and post-procedure. Levels of RANTES (regulated on activation, normal T cell expressed, and secreted) were significantly lower in responders, both at baseline (12.821 ± 3646 vs. 15,102 ± 3069 pg/mL) and at 30 days (13,941 ± 3098 vs. 16,108 ± 2209 pg/mL) (*p* = 0.037), without significant variation throughout the time. ROC curve analysis showed an area under the curve of 0.701, 95% CI = 0.567–0.834 (*p* = 0.01). A RANTES level ≤ 15,496 pg/mL showed the best overall sensitivity (57.1%) and specificity (85.7%) for determining response to RDN. Even though late-responders (R1Y) continued to present with lower levels of RANTES at every time point, this difference did not reach statistical significance (Figure 1). There was a trend for higher IL-6 levels in the overall cohort of responders at baseline, however this did not reach statistical significance. The principal component analysis for the distribution of cytokines, chemokines, and growth factors at the four analyzed times (panel A) and at baseline (panel C) is illustrated in Figure 2. Panels B and D illustrate the overall and baseline heatmaps representing clustering of multivariate data.

### 3.3. Dynamics of Soluble Factors in Renal Denervation Patients

**Figure 3:**
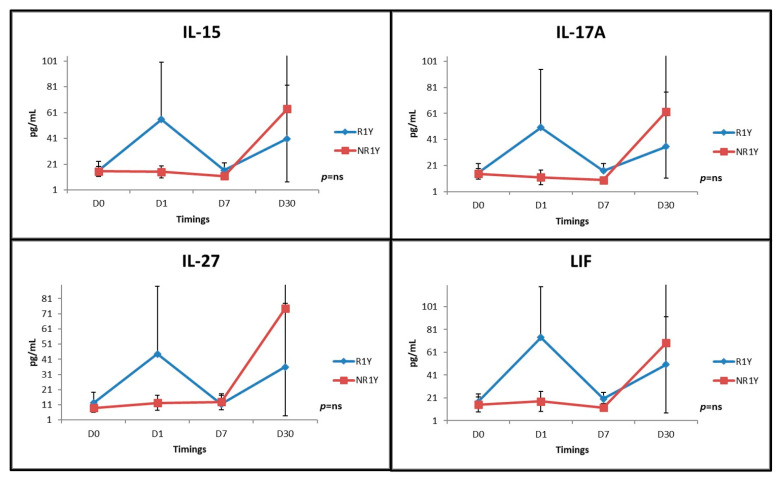
Significant variation in time was observed when analyzing IL-15, IL-27, IL-17A, and LIF at four time points (D0, D1, D7, and D30), with responders presenting an acute rise 24 h after RDN. Panels indicate values between R1Y and NR1Y. The same pattern is maintained when analyzing response at six months (not shown). Data are presented as mean ± standard deviation. LIF—leukemia inhibitor factor; R1Y—responders at one year; NR1Y—non-responders at one year.

Although absolute levels of other analyzed cytokines were not statistically different between groups, a pattern was evident when analyzing their behavior. IL-15, IL 17A, IL 27, and leukemia inhibitory factor (LIF) had significant variability through time, with responders presenting with an acute rise at D1 (24 h after RDN) (Figure 3).

Furthermore, other biomarkers known to be associated with cardiovascular disease [15,16] were also quantified. Both NR (*p* = 0.02) and NR1Y (*p* < 0.001) had higher levels of baseline glycated hemoglobin and, in NR, levels of NT pro-BNP were significantly higher (*p* = 0.005), a difference not observed at one year follow-up. No other significant differences were found regarding renal function, LDL levels, C-reactive protein, lipoprotein A, and fibrinogen (Table 1).

### 3.4. Soluble Factors Were Correlated with Double-Negative T-Cells in Responders

**Figure 4:**
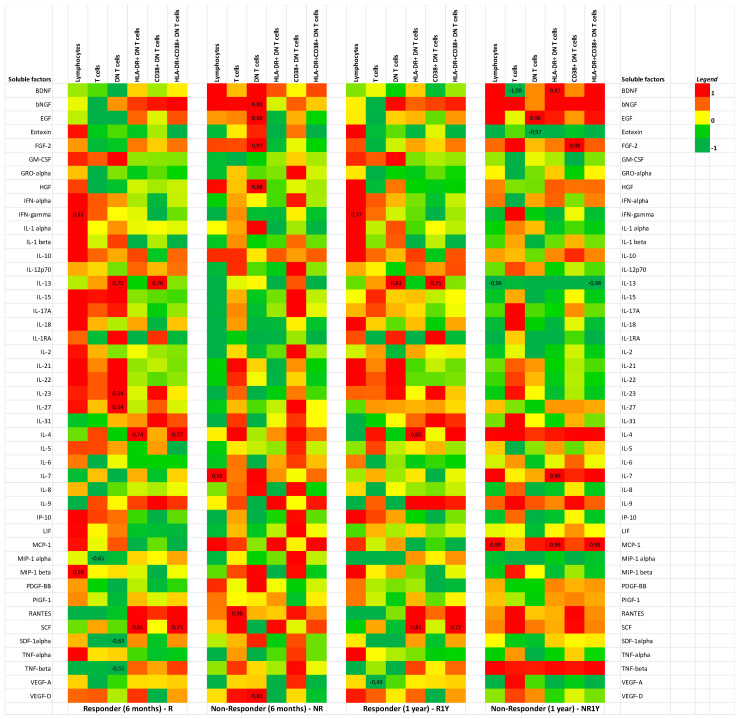
Correlation between soluble factors and DN T cells in responders. Panels present heatmaps of soluble factors according to response (R/NR—6 months; R1Y/NR1Y—one year. Pearson’s correlation was used to explore the association of soluble factors and cell populations indicated at the top of each block. Pearson’s values are indicated in squares and correspond to significant *p* values (<0.05). LIF—leukemia inhibitor factor; R1Y—responders at one year; NR1Y—non-responders at one year.

Previously, our research group has determined that HLA-DR+ DN T cells were significantly lower in R1Y with this difference being most evident before the procedure [14]. In the present research, we analyzed the baseline correlation between soluble factors and DN T cells in responders at six months and one year. IL-13 positively correlated with DN and CD38+ DN T cells in R (r = 0.75, *p* = 0.002; r = 0.76, *p* = 0.003) and R1Y (r = 0.61, *p* = 0.009; r = 0.75, *p* = 0.001). IL-27 positively correlated with DN T cells in R (r = 0.54, *p* = 0.04). Positive correlations were also evident between the cytokine IL-4 and HLA-DR+ and CD38+ DN T cells. A negative correlation was found between the cytokines stromal cell-derived factor 1α (SDF-1α) and tumor necrosis factor α (TNF-α) and DN T cells in R. (Figure 4).

## 4. Discussion

Our study has uncovered important findings regarding the dynamics of cytokines, chemokines, and growth factors in patients with resistant HT. The mechanisms that contribute to the development of HT are complex and involve renal deregulation, endothelial dysfunction, and unbalanced central and autonomic nervous systems. Inflammation is directly linked to the development of HT and is a complicated process involving multiple cell types and secreted pro- and anti-inflammatory substances.

Firstly, we demonstrated that responders presented with lower levels of RANTES than non-responders. Knowledge about circulating concentrations of this chemokine in patients with HT is still insufficiently documented. RANTES (also called CCL5, CC-motif ligand 5) is a soluble pro-inflammatory chemokine secreted by several cell types such as activated T cells, fibroblasts, endothelial cells, smooth muscle cells, glial cells, mesangial cells, and platelets [17,18], which is highly expressed in atheroma and has been implicated in the pathophysiology of atherosclerosis [19]. Data regarding the role of RANTES in atherosclerosis and plaque vulnerability is controversial. Studies that included patients with acute coronary syndromes have found elevated RANTES levels [20], while others have shown that low RANTES levels were independently predictive of adverse outcomes in chronic stable disease [21]. RANTES is known to be a very potent chemo-attractant of T-cells, monocytes, and macrophages, and it is possible that its higher levels may be associated with cellular infiltration and hence end-organ damage. Yun et al. [22] observed that angiotensin II, mediated by 12-lipoxygenase, inhibited RANTES expression in spontaneous hypertensive rats’ vascular smooth muscle cells, through the activation of AT1 and AT2 receptors. As the SNS is directly linked to RAAS [23], we may deduce that lower levels of RANTES are associated with higher levels of angiotensin II and therefore with an overactivated SNS, making these patients more prone to a RDN response. Our study also demonstrated that, in spite of lower levels of RANTES that persisted at one year follow-up, this difference was not statistically significant, probably due to the low number of non-responders at this stage. In our study, even though responders had lower levels of RANTES, they were not suppressed and, in fact, were above 10.000 pg/mL. Previously, a protective role of RANTES for the kidney, that appears tissue-specific, has been described by Rudemiller et al. [24]. The authors observed that RANTES deficiency led to exaggerated hypertensive renal damage through macrophage accumulation, up-regulation of TNF and IL-1β and renal parenchyma matrix deposition, and fibrosis. In light of this information and the results of our study, we hypothesize that RANTES continued to exert its protective role in the kidney, in both responders and non-responders, as renal function was within normal values in the overall cohort.

Secondly, in our study several cytokines had significant variability at the evaluated time points. It was possible to depict a pattern regarding the levels of IL-15, IL-27, IL-17A, and LIF, as responders presented peak levels 24 h after RDN. Although there was a trend towards higher IL-6 values at baseline in responders, this difference was not significant. Radiofrequency energy is directly applied to the renal wall and the vessel temperature is increased to destroy the nervous sympathetic terminals wrapped around the renal artery. Despite the fact that current research shows there is no luminal damage to the vessel at a medium-term follow-up, there is evidence of endothelial edema, vessel spasm and intraluminal thrombus formation immediately after the procedure, that rapidly heals [25]. These alterations are likely to provoke an inflammatory reaction, probably directly related to the amount of induced nerve damage. As such, the acute rise of pro-inflammatory cytokines in responders, 24 h after RDN, may indicate a successful nerve ablation. Lee et al. [26] investigated the changes of early inflammatory biomarkers following RDN and verified that several pro-inflammatory cytokines (IL-1β, IL-18, IL-6, and TNF-α), and also caspase-1 activity, and NLRP3 expression, increased immediately after RDN and then recovered two weeks afterwards, suggesting a self-limited inflammatory response to RDN. There is evidence linking RANTES with metabolic syndrome and IL-6 [27], which in turn is directly related to endothelial dysfunction and therefore to an increased risk of HT and cardiovascular events, possibly having an important role in the progression of atherosclerosis [28]. Lang et al. examined several renal and inflammatory parameters in resistant HT treated with RDN and observed that IL-6 and white blood cell count significantly decreased 6 and 12 months after the procedure. At baseline, a trend towards higher IL-6 values was evident but the difference was not statistically significant and thus not useful to predict response in the studied cohort, a finding corroborated by our research [29]. Dörr et al. also determined IL-6 levels in sixty patients treated with RDN and observed that IL-6 diminished significantly after the procedure, hypothesizing a beneficial effect of RDN on cardiovascular inflammation [30]. Some other studies have investigated the effects of RDN on pro-inflammatory cytokine environment. Hilderman et al. verified that TNF and IL-1b values were significantly lower one day after RDN, and that IL-10, an anti-inflammatory cytokine, rose significantly after the procedure, compared to a control group. These differences, however, were less evident in the medium-term follow-up, suggesting a transitory immuno-modulatory effect [31]. IL-15 is a pro-inflammatory cytokine which is a potent chemo-attractant for T cells and is expressed predominantly by macrophages. It has been shown that increased levels of IL-15 are associated with progression of atherosclerotic disease and more severe degrees of HT [32]. IL-27 belongs to the IL-12 family and is considered to have pro- and anti-inflammatory properties. It induces interferon-γ (IFN-γ), IL-1, and TNF-α production, but may also induce IL-10 production by T-cells [33]. IL-17A is a well-known effector cytokine produced by Th17 cells and is involved in tissue inflammation in several chronic inflammatory diseases, including HT [34]. LIF is a member of the IL-6 family and activates pathways that promote cardio-protection, both in the acute and chronic contexts, by protecting against oxidative stress and cell death and by stimulating differentiation of cardiac stem cells into endothelial cells and neovascularization, post-MI [35]. The fact that, in our research, pro- and anti-inflammatory cytokines peaked after 24 h is a very interesting finding and we hypothesize that the provoked numbness of the SNS allowed for anti-inflammatory pathways to counteract the acute inflammatory process. This is in line with the findings of Lee et al. [26], who observed an increase in inflammatory cytokines IL-1β, IL-18, IL-6, and TNF-α, and anti-inflammatory cytokine IL-10, immediately after RDN and then a decrease in week 2 of the follow-up.

SNS hyper-activation associated with profuse inflammatory response is also triggered by ischemia/reperfusion (I/R) injury. This association has been examined by Sun et al. [36] in translational research, in which a group of patients with ST-elevation acute myocardial infarction (MI) and a group of mice were treated with surgical RDN and I/R injury through left anterior descending coronary artery suture or ligation. They observed that MI patients had elevated norepinephrine and leucocyte plasma levels. Denervated mice showed a reduction in neutrophils and macrophages in blood and myocardium, associated with a significant decrease in IL-1, IL-6, and TNF-α. The authors concluded that a link appears to exist between the SNS and the inflammatory response following I/R injury, identifying RDN as a potential therapeutic strategy in this setting.

Thirdly, in our study there was a positive correlation between levels of IL-13 and IL-4 at baseline (D0) and DN, HLA-DR+, and CD38+ DN T cells, in responders. IL-13 and IL-4 are both anti-inflammatory cytokines. Studies have shown that these cytokines may stimulate the pressor response but do not contribute to the enhanced sympathetic nerve activity usually present in resistant HT patients [37]. Therefore, they are prone to be reduced in the responders, who presumably have a hyperactivated SNS and thus clinically respond to RDN. There was also a negative correlation between the pro-inflammatory cytokines SDF-1α and TNF-1α, and DN T cells, in responders (D0). It has been demonstrated that SDF-1α is involved in the migration of cancer cells and it also increased in the serum and aorta of patients with Ag II-induced HT [38]. Therefore, these findings confirm the pro-inflammatory milieu associated with refractory HT.

Finally, several parameters were evaluated to assess safety and for risk stratification. Glycated hemoglobin was significantly higher in non-responders, even though the number of diabetic patients was similar between groups, implying that poorly controlled glycaemia may adversely affect response to RDN. There is evidence connecting the activation of renal sympathetic nerves to insulin resistance and impairment of glucose metabolism [39,40]. However, the method by which RDN may impact glucose and insulin metabolism remains unknown, and complicated mechanisms involving the SNS, RAAS, vascular α-adrenergic tone, and improved glucose transport to skeletal muscle may be potential explanations. The Dreams study aimed to study the impact of RDN in patients with metabolic syndrome and concluded that RDN did not lead to a significant improvement of insulin sensitivity [41]. Thus, more clinical trials are needed before any conclusion can be drawn regarding this matter.

## 5. Limitations

This is a single-center, non-randomized study, with a relatively small sample size and no control group. Larger cohort studies will be necessary to confirm these results.

## 6. Conclusions

Our study analyzed the behavior of several cytokines in patients treated with renal denervation and identified RANTES as a potential predictor of a response. Additionally, some pro- and anti-inflammatory cytokines peaked 24 h after the procedure and decreased one week later. Our group has previously showed that HLA-DR+ double-negative T cells were significantly lower in responders. There was a positive correlation between IL-13, -27, and -4, and double-negative T cells, and a negative correlation between the latter and SDF-1α and TNF-α at baseline, probably indicating enhanced inflammation at baseline in this group of patients. As a final conclusion, we can confirm that renal denervation effectively lowered blood pressure in the majority of treated patients, without safety concerns.

## 7. Highlights

- The role of the immune system in the pathophysiology of hypertensive patients treated with renal denervation is not clear.
- RANTES is a potential predictor of response to renal denervation.
- IL-15, IL-27, IL-17A, and LIF increased 24 h after renal denervation in responders and decreased one week later.
- Pro-inflammatory cytokines negatively correlated with double-negative T cells (shown to be reduced in responders).
- Renal denervation effectively lowered blood pressure in the majority of patients.

## Figures and Tables

**Table 1:** Baseline clinical, biochemical and procedural characteristics. Ambulatory blood pressure monitoring behavior, from baseline to one year follow-up.

	R (*n* = 14)	NR (*n* = 7)	*p* Value	R1Y (*n* = 17)	NR1Y (*n* = 4)	*p* Value
Age in Y (mean ± SD)	61 ± 10	55 ± 13.5	ns	59.8 ± 11.8	55.5 ± 9.5	ns
Diagnosis of HT in Y (mean ± SD)	16.3 ± 9.3	17 ± 5.9	ns	16.6 ± 8.6	16.2 ± 7.5	ns
Female sex (%)	21.4	57.1	ns	35.3	25	ns
Dyslipidemia (%)	85.7	100	ns	88.2	100	ns
Type 2 diabetes (%)	42.9	57.1	ns	47.1	50	ns
Active smoking (%)	14.3	42.9	ns	11.8	75	**0.008**
Sleep apnea (%)	57.1	57.1	ns	58.8	50	ns
Number of HT drugs (*n* ± SD)	5.1 ± 1.4	5.4 ± 0.5	ns	5.2 ± 1.3	5.2 ± 0.5	ns
On spironolactone (%)	57.1	42.9	ns	52.9	50	ns
Isolated HT (%)	14.3	14.3	ns	17.6	0	ns
BMI (Kg/m^2^)	29.4 ± 3.8	30.2 ± 4.3	ns	30.1 ± 3.8	27.8 ± 4.3	ns
***Biochemical profile at baseline***						
HBA1c (%)	5.9 ± 0.75	7 ± 2.5	**0.02**	5.9 ± 0.7	7.7 ± 3.4	**<0.001**
NT pro-BNP (pg/mL)	165 ± 265	1037 ± 2478	**0.005**	541 ± 1594	94.7 ± 62.2	ns
LpA (mg/dL)	42.3 ± 46	30 ± 45.2	ns	37.6 ± 42.7	40.6 ± 60.9	ns
Fibrinogen (mg/dL)	278.1 ± 59.1	271.2 ± 76.5	ns	282 ± 65	243.7 ± 44.8	ns
Creatinin (mg/dL)	0.97 ± 0.2	0.76 ± 0.2	ns	0.9 ± 0.2	0.76 ± 0.2	ns
Cystatin C (mg/L)	0.97 ± 0.2	0.85 ± 0.2	ns	0.95 ± 0.2	0.8 ± 0.1	ns
LDL col (mg/dL)	98 ± 23	118 ± 26	ns	102 ± 25	116 ± 28	ns
PCR (mg/dL)	0.83 ± 0.97	1.2 ± 2.2	ns	1.1 ± 1.6	0.4 ± 0.3	ns
***Baseline***						
ABPM systolic BP (mmHg)	154 ± 12.3	153.4 ± 12.9	ns	154.8 ± 12.5	149.7 ± 11.6	ns
ABPM diastolic BP (mmHg)	87.1 ± 14.3	92.7 ± 15.6	ns	89 ± 15.3	88.7 ± 13.3	ns
HR (bpm)	70.6 ± 9.1	73.3 ± 13.9	ns	72.3 ± 9.8	68 ± 15	ns
E/E’ ratio	10.6 ± 4.8	15 ± 3.6	ns	11.4 ± 5.2	13.8 ± 3.5	ns
***Six Months***						
ABPM systolic BP (mmHg)	133 ± 11	161 ± 9.7	**<0.001**	138.6 ± 16.6	158.2 ± 7.4	**0.004**
ABPM diastolic BP (mmHg)	77.1 ± 11.2	95.1 ± 15.8	**0.007**	81.3 ± 15	90.5 ± 13.7	ns
HR (bpm)	68.8 ± 7.3	75.8 ± 10.8	0.09	71.3 ± 8.7	70.5 ± 11.7	ns
E/E’ ratio	12.8 ± 6.3	12.3 ± 2.8	ns	12.6 ± 5.2	12.5 ± 3.4	ns
***One year***						
ABPM systolic BP (mmHg)	135.9 ± 16.2	149.3 ± 20.2	ns	135.6 ± 15.8	160.5 ± 15	**0.03**
ABPM diastolic BP (mmHg)	77.1 ± 11.2	88.4 ± 14.3	ns	77.5 ± 10.5	94.5 ± 15.8	ns
HR (bpm)	67.8 ± 8.1	74.4 ± 12	ns	69.4 ± 8.3	72.5 ± 16.3	ns
**Total ablations (*n*)**	23.8.6 ± 8.3	22.4 ± 7.3	ns	23.9 ± 8.5	20.7 ± 4	ns
**Drop in ABPM systolic BP at 6 months (mmHg)**	21.1 ± 13.3	−7.6 ± 10.6	**<0.001**	16.2 ± 16.6	−8.5 ± 11.9	**0.013**
**Drop in ABPM systolic BP at 1 year (mmHg)**	18.1 ± 11.5	4 ± 21.5	ns	19.2 ± 12.2	−10.7 ± 6.7	**<0.001**

Legend—R: responder; NR: non-responder; R1Y: responder at one year; NR1Y: non-responder at one year; Y: years; HT: hypertension; BMI: body mass index; ABPM: ambulatory pressure monitoring; BP: blood pressure; HR: heart rate; HBA1c: glycated hemoglobin; LpA: lipoprotein A; Col: cholesterol; PCR: C-reactive protein. Results are displayed as mean ± standard deviation (SD).

## Data Availability

Not applicable.

## Abbreviations

RDN	renal denervation
BP	blood pressure
HT	hypertension
SNS	sympathetic nervous system
ABPM	ambulatory blood pressure monitoring
